# Comparative Analysis of Black Chokeberry (*Aronia melanocarpa* L.) Fruit, Leaves, and Pomace for Their Phytochemical Composition, Antioxidant Potential, and Polyphenol Bioaccessibility

**DOI:** 10.3390/foods13121856

**Published:** 2024-06-13

**Authors:** Mihaela Saracila, Arabela Elena Untea, Alexandra Gabriela Oancea, Iulia Varzaru, Petru Alexandru Vlaicu

**Affiliations:** Feed and Food Quality Department, National Research and Development Institute for Animal Biology and Nutrition, Balotesti, 077015 Ilfov, Romania; arabela.untea@ibna.ro (A.E.U.); alexandra.oancea@ibna.ro (A.G.O.); alexandru.vlaicu@outlook.com (P.A.V.)

**Keywords:** black chokeberry, phytochemicals, polyphenols bioaccessibility, in vitro gastrointestinal digestion, antioxidant potential

## Abstract

The study aims to compare the nutrient composition, antioxidant potential, and polyphenol bioaccessibility of the fruit, leaves, and pomace of black chokeberry. Phytochemical characterization, antioxidant activity, and the effect of in vitro gastrointestinal digestion on the individual phenolic compounds of fruit, leaves, and pomace of black chokeberry were assessed. Results showed that leaves had a higher content of polyphenols (61.06 mg GAE/g dw), flavonoids (8.47 mg QE/g), and tocopherols (1172.20 mg/kg) than fruit (27.99 mg GAE/g dw polyphenols, 5.23 mg QE/g flavonoids, 38.48 mg/kg tocopherols) and pomace (22.94 mg GAE/g dw polyphenols, 1.89 mg QE/g flavonoids and 157.19 mg/kg tocopherols), with superior in vitro antioxidant activity. Chlorogenic acids were the dominant phenolic compounds in black chokeberry undigested samples (2.713 mg/g in fruit, 17.954 mg/g in leaves, and 1.415 mg/g in pomace) but are poorly absorbed (bioaccessibility index in intestinal phase of 28.84% for fruit, 8.81% for leaves, and 31.90% for pomace). Hydroxybenzoic acids were highly stable in leaves and fruit during simulated digestion and had high bioaccessibility. In conclusion, residues from black chokeberry processing are also valuable sources of bioactive compounds, but the pomace had higher polyphenol bioaccessibility than leaves and might be a promising supplement for the food industry.

## 1. Introduction

Berries are becoming more and more popular worldwide due to their high content of essential vitamins, minerals, dietary fiber, and polyphenolic compounds, which are believed to provide numerous potential health benefits [1]. Human nutritionists recommend including berries in dietary guidelines across the globe because of their ability to prevent various diseases and disorders [2]. One of the many types of berries is the black chokeberry (*Aronia melanocarpa* L.). The black chokeberry, a perennial shrub indigenous to eastern North America, was introduced to Europe in the 20th century [3]. The leaves are used in traditional medicine as anti-inflammatory, antiviral, antibacterial, and anti-proliferative agents [4,5,6]. The fruits and leaves of black chokeberry are known to have a positive impact on health due to their various bioactive components, which include vitamins, minerals, and polyphenolic compounds [7]. Chokeberries are a rich source of polyphenols, including anthocyanins, proanthocyanidins, flavonols, flavanols, proanthocyanidins, and phenolic acids [7]. These biofactors determine the high bioactivity of chokeberries, and the main polyphenolic components of these fruits possess numerous health benefits [3]. Chokeberry fruits are not commonly consumed directly due to their astringent taste caused by polyphenols and tannins [8]. Rather, it is mostly used to make jams, juices, syrups, and other similar products. The by-products resulting after fruit processing, such as pomace, are also being utilized more frequently. Chokeberry pomace contains around 28–35% skin, 60–70% seeds, and around 10% pulp [9]. Pomace is a good source of polyphenols, vitamins, and dietary fiber, and it is low in calories [10]. The polyphenol content in pomace ranges from 3100 to 6300 mg/100 g dry matter (DM) [11], with higher levels in seedless fractions compared to those with seeds [12]. It was found that the content of procyanidins in pomace is higher than in juices and fresh fruit [7]. As a result, it has the potential to be an excellent source of natural antioxidants [13]. Despite numerous research articles published on the utilization of by-products from the agro-industry, including fruit pomace, the juice-pressing residues of certain fruits are still under investigation [14]. Pomace that has been dried and powdered can serve as an effective ingredient in a range of products, including bakery items, confectioneries, functional goods, and teas, as reported by Raczkowska et al. [8].

The nutraceuticals and supplements markets are experiencing rapid growth. In the quest for effective sources of these products, scientists are increasingly turning to materials traditionally regarded as waste, such as pomace, seeds, and leaves [15]. This trend aligns with the zero-waste initiative set forth by the European Union to be achieved by 2025 and supports the Sustainable Development Goals (SDGs). Recent research has highlighted fruit leaves as particularly valuable, being rich in bioactive compounds that confer numerous health benefits [16,17].

The demand for black chokeberries is increasing due to their value as a source of food and raw materials for the food industry, leading to growth in its production. However, the leaves of the plant are often discarded as waste despite containing important quantities of polyphenols. It was reported that the total phenolic content of leaf extracts can vary between 1946 and 9148 mg/100 g, with higher levels found in more mature leaves [18]. Currently, researchers and the food industry are focused on developing functional food formulations and nutraceuticals, making this study align with global trends [8]. Chokeberry leaves and pomace have the potential to be considered components for designing antioxidant-rich foods and nutraceuticals. Extensive studies are required to assess the antioxidant properties and polyphenols kinetics of black chokeberry during digestion, similar to other natural plants and medicinal products. There is insufficient scientific literature available on the bioaccessibility of polyphenols found in black chokeberry fruit, pomace, and leaves despite considerable research conducted on them. This information would be beneficial in determining the extent to which the contained polyphenols in fruit, leaves and pomace are bioaccessible and can exert biological effects.

This study aims to provide analytical data regarding nutrient composition (proximate, mineral composition, fatty acids profile), antioxidant potential (lipo and hydro soluble compounds, antioxidant activity), and in vitro gastrointestinal (GI) digestion of different black chokeberry samples (fruits, leaves, and pomace).

## 2. Materials and Methods

### 2.1. Materials

The material used for the study consisted of fruit, leaves, and pomace from black chokeberry (*A. melanocarpa* L.) and were collected from a local producer of Aronia juice in Dambovita county, (44°53′ N 25°28′ E), Romania. Samples of fruits and leaves were collected together in the stage of full physiological maturity in August 2023, when the color of the fruits was dark purple. The collected material was dried and ground into a fine powder using a Grindomix GM 200 mill (Retsch, Haan, Germany) using a 0.5 mm sieve. Average samples were subsequently formed and carefully stored in darkness until the determinations were carried out.

### 2.2. Chemical Composition

#### 2.2.1. Proximate Analysis and Mineral Composition

Dry matter (984.25), ash (925.51), protein (950.36), fat (935.38), and dietary fiber (985.29) content of samples were determined by standard methods defined by the Association Official of Analytical Chemists (Washington, DC, USA) [19]. The dry matter was determined using a BMT oven, model ECOCELL Blueline Comfort, from Nuremberg, Germany. Ash was analyzed using a Nabertherm calcination furnace from Nabertherm GmbH in Lilienthal, Germany. Crude protein was determined using a Kjeltec auto 1030 from Tecator Instruments in Höganäs, Sweden, while crude fat was determined with a Soxtec 2055 from Foss Tecator, also in Höganäs, Sweden. Finally, crude fiber was determined using a Fibertec 2010 System from Foss Tecator in Höganäs, Sweden.

The content of assimilable carbohydrates in the studied samples was calculated using the following equation [8]:

Assimilable carbohydrates = dry matter − (dietary fibre + fat + protein + ash)(1)

The content of copper, iron, manganese, and zinc in samples was determined by flame atomic absorption spectrometer (Thermo Electron SOLAAR M6 Dual Zeeman Comfort, Cambridge, UK) after microwave digestion (Berghof, Eningen, Germany) according to Untea et al.’s [20] method.

#### 2.2.2. Fatty Acids

The extraction of fatty acids was performed in petroleum ether and followed the steps according to AOAC [19]. Extracted fat was mixed with 50 mL of acidified methanol and boiled under reflux with a water bath (FALC WB-U6, FALC Instruments, Treviglio, Italy) for 25–30 min. After cooling, it was mixed with distilled water and hexane, stirred, and transferred to a separation funnel. The organic layer was rinsed with distilled water, dried using anhydrous Na_2_SO_4_, and concentrated using a rotary evaporator. The residue was dissolved in hexane and placed in a volumetric flask for analysis. The composition of fatty acid was assessed with a PerkinElmer Clarus 500 gas chromatograph (Waltham, MA, USA). This system was equipped with a flame ionization detector (FID) and a capillary separation column containing a high polar stationary phase TRACE TR-Fame, as described elsewhere [21]. The amount of each fatty acid was expressed as g/100 g fatty acid methyl esters (FAME).

#### 2.2.3. Extraction Procedure

Extraction for total phenolic content, flavonoids, and antioxidant activity assays was performed in methanol (80%) according to the study published previously by Untea et al. [22]. To obtain extracts, the samples were placed on a rotary shaker in a dark environment for 24 h and centrifuged (1500× *g*, 10 min) with a refrigerated benchtop centrifuge (Sigma 3–16 KL) to obtain the supernatant.

Extraction for the fat-soluble compounds like xanthophylls and vitamin E from the samples was performed with a method previously described by Varzaru et al. [23]. First, a preparation technique was used that included a saponification step using a reflux water bath (FALC WB-U6, FALC Instruments). This step was carried out using a solution of ethanolic potassium hydroxide in a water bath at 80 °C for 30 min. Then, the extraction was performed using petroleum ether.

#### 2.2.4. Total Phenolic Content

The total phenolic content (TPC) was measured with Folin–Ciocalteu’s reagent as originally described by Untea et al. [22]. Absorbance was recorded at 732 nm wavelength with a microplate reader, Varioskan Lux (Thermo Fischer Scientific, Waltham, MA, USA). The results were obtained with a previously constructed calibration curve (R^2^ = 0.9993), and mean values of TPC of prepared samples were expressed as milligrams of gallic acid equivalents (GAE) per g of the dry weight of the sample (mg GAE/g dw).

#### 2.2.5. Total Flavonoid Content

Total flavonoid content was determined using the method described by Zou et al. [24]. The absorbance of samples was measured against the blank at 410 nm with a Varioskan Lux microplate reader (Thermo Fischer Scientific, Waltham, MA, USA). The calibration curve was generated through the utilization of quercetin as the standard (R^2^ = 0.9972). The flavonoid content was quantified in terms of milligrams of Quercetin equivalents (QE)/per g of dry weight of the sample (mg QE/g dw).

#### 2.2.6. Xanthophylls Content

Xanthophylls (lutein (95%), zeaxanthin (95%), astaxanthin (97%), canthaxanthin (95%), Sigma-Aldrich (St. Louis, MO, USA)) were analyzed according to Varzaru et al. [25] using an HPLC series 200 (Perkin Elmer, Shelton, CT, USA) equipped with a UV detector (detection at 450 nm). Reversed-phase column C18 (250 × 4.60 mm, 5 μm) (Nucleodur, Macherey-Nagel, Duren, Germany) was used, and the method was performed in isocratic conditions at a flow rate of 0.5 mL/min at 25 °C, with a mobile phase of 10% water, 15% methanol, and 75% acetone. The results were expressed as mg/kg.

#### 2.2.7. Vitamin E Analysis

The isomers of vitamin E were identified with a liquid chromatograph, specifically the Vanquish model manufactured by the Thermo-Electron Corporation in Waltham, MA, USA. A PDA-UV detector was utilized at a wavelength of 292 nm, following the methodology described previously by Varzaru et al. [25]. The results were expressed as mg/kg. The C18 reversed-phase column (5 µm, 250 × 4.60 mm i.d.) (Thermo-Electron Corporation, Waltham, MA, USA) was used with a mobile phase of 96% methanol and 4% water at a flow rate of 1.5 mL min^−1^.

#### 2.2.8. Identification and Quantification of Polyphenols

In order to determine individual phenolic compounds within a sample of vegetal powder, the extraction procedure was conducted according to the parameters outlined in Untea et al. [16]. Specifically, 0.5 g of the sample was mixed with 10 mL of an extraction solvent consisting of water/methanol/acetic acid (69:30:1, *v*/*v*/*v*). Following this, the mixture was placed into screw-cap test tubes and subsequently incubated at 50 °C for 60 min in a shaking water bath. Thereafter, the samples were centrifuged at 4000 rpm for 15 min. For the qualitative and quantitative determination of polyphenolic compounds using the liquid chromatographic method, the procedure was as described by Untea et al. [16]. The polyphenols were separated utilizing a Vanquish Core HPLC system, which was outfitted with a DAD that was manufactured by Thermo Fisher Scientific in Bremen, Germany, at a temperature of 25 °C. Furthermore, a BDS HyperSil C18 column, measuring 250 × 4 mm with a particle size of 5 µm, from the same manufacturer in Bremen, Germany, was employed for the separation procedure. The mobile phase consisted of (A) acetic acid (1%) in distilled water (*v*/*v*), (B) methanol, and (C) acetonitrile. The flow rate was set at 0.5 mL/min. Polyphenols such as ellagic acid, syringic acid, epicatechin, 4-hydroxy-3-methoxy-cinnamic acid, rutin, vanillic acid, 3-hydroxybenzoic acid, protocatechuic acid, caffeic acid, coumaric acid, epigallocatechin, catechin, quercetin, and resveratrol were purchased from Sigma-Aldrich (St. Louis, MO, USA). They were utilized for the identification and quantification of polyphenolic compounds. Ferulic acid and chlorogenic acid were purchased from the European Pharmacopoeia (EP).

The gradient used in the experiment involved the steps presented in Table 1.

### 2.3. Antioxidant Activity

The antioxidant activity was evaluated using four methods, based on the samples’ phytochemicals to scavenge stable diphenyl-picrylhydrazyl radical (DPPH^•^), 2,20-azino-bis 3-ethylbenzo-thiazoline-6-sulfonic acid radical cation (ABTS^•+^) and to evaluate their iron chelating power as well as total antioxidant activity of plant extracts (phosphomolybdenum method).

#### 2.3.1. Determination of Antioxidant Activity Using the 2,2-Diphenyl-1-picrylhydrazyl (DPPH) Radical Scavenging Method

DPPH^•^ scavenging activity of samples was determined by a slightly modified spectrophotometric method of Qwele et al. [26]. The quantification of a decrease in absorbance at 517 nm was carried out using a microplate reader known as Varioskan Lux, manufactured by Thermo Fischer Scientific, Waltham, MA, USA. The calibration curve was prepared by plotting the absorbance at 517 nm versus different concentrations of 6-hydroxy-2,5,7,8-tetramethylchroman-2-carboxylic acid (Trolox). The antioxidant activity of each sample is expressed as millimoles of Trolox equivalents (TE) per kilogram of sample. The percentage inhibition was calculated using the following formula:

% Inhibition = (AC − AS) × 100/AC(2)

#### 2.3.2. Determination of Antioxidant Activity Using 2,2′-Azinobis-3-Ethylbenzthiazolin-6-Sulfonic Acid (ABTS) Free Radical Scavenging Method

The scavenging activity of samples against ABTS^•+^ was determined using a method that relied on the interaction between an antioxidant and pre-generated ABTS^•+^ radical cation in accordance with the guidelines set forth by Saracila et al. [27]. The absorbance of samples against a blank was read at 734 nm using a microplate reader Varioskan Lux (Thermo Fischer Scientific Waltham, MA, USA). The calibration curve was prepared by plotting the absorbance at 734 nm versus different concentrations of 6-hydroxy-2,5,7,8-tetramethylchroman-2-carboxylic acid (Trolox) (R^2^= 0.9929). The results were quantified as millimoles Trolox equivalents (TE) per kilogram of sample. The percentage inhibition was calculated using the following formula:

% Inhibition = (AC − AS) × 100/AC(3)

#### 2.3.3. Evaluation of Antioxidant Activity by Iron Chelating Power

The methodology for the determination of the chelating effect on ferrous ions was adapted from the method described by Sabeena Farvin et al. [28] and Santos et al. [29] with slight modifications [27]. The Varioskan Lux microplate reader (Thermo Fisher Scientific, Waltham, MA, USA) was used to measure the absorbance of the purple color of the complex formed when an extract competed with ferrozine for ferrous ions against the blank at 562 nm. The calibration curve was prepared by plotting the absorbance at 562 nm versus different concentrations of EDTA (R^2^ = 0.9971). The chelating effect on ferrous ions was quantified as mg EDTA equivalents per gram of the sample (mg EDTA equiv./g). The percentage inhibition was calculated using the following formula:

% Inhibition = (AC − AS) × 100/AC(4)

#### 2.3.4. Evaluation of Total Antioxidant Activity by Phosphomolybdenum Method

The total antioxidant activity of Aronia samples was measured using the phosphate-molybdenum method according to Prieto et al. [30] with slight modification [22]. The absorbance was recorded at 695 nm with a Varioskan Lux microplate reader (Thermo Fischer Scientific, Waltham, MA, USA). The results were expressed as mmol ascorbic acid equivalent/kg, which are commonly used units for expressing the antioxidant activity of samples.

### 2.4. In Vitro Gastrointestinal (GI) Digestion of Black Chokeberry Samples

**Figure 1:**
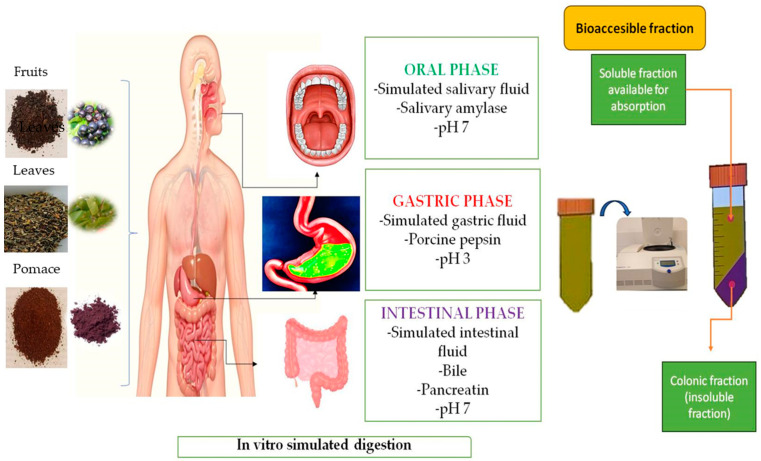
Schematic and design protocol of the in vitro simulated gastrointestinal digestion on phenolic components of black chokeberry samples (fruit, leaves, and pomace).

The current study aims to evaluate the in vitro gastrointestinal digestion of Aronia samples using the static method protocol INFOGEST, according to Minekus et al. [31]. The protocol proved effective in simulating the salivary, gastric, and intestinal phases of digestion. The results of this study may provide insights into the digestive properties of Aronia samples and their potential health benefits. Figure 1 shows the flow diagram of the static in vitro model used for black chokeberry fruit, leaves, and pomace digestion and the analysis of individual polyphenols. The salivary phase was simulated by mixing 5 g of sample (fruit, leaves, pomace) with 3.5 mL of simulated salivary fluid (SSF) electrolyte stock solution and mincing the mixture. Subsequently, 0.5 mL of salivary a-amylase solution of 1500 U/mL made up of SSF electrolyte stock solution was added to the mixture, followed by 25 µL of 0.3 M CaCl_2_ and 975 µL of distilled water. The mixture was then adequately mixed and incubated at 37 °C for 2 min. To simulate the gastric phase, the oral bolus derived from the simulated oral phase was mixed with 7.5 mL of the simulated gastric fluid (SGF). Then, 1.6 mL of a porcine pepsin solution was added to the mixture, resulting in a final concentration of 2000 U/mL. Finally, 5 µL of 0.3 M calcium chloride solution was added. The final volume of the solution was adjusted to 10 mL by adding distilled water after the pH was lowered to 3 with 6 M HCl. The solution was subsequently placed in an incubator at a temperature of 37 °C for 2 h. For the intestinal phase simulation, the gastric chyme was mixed with 11 mL of simulated intestinal fluid (SIF), 5 mL of pancreatin solution (800 U/mL), 2.5 mL of 160 mM bile salts, and 40 µL of 0.3 M CaCl_2_ and 1 M NaOH to reach pH 7.0. The samples were then incubated at 37 °C for 2 h. After each stage of the in vitro digestion simulation, the samples were subjected to a process of centrifugation. To execute the procedure, we utilized a laboratory-grade refrigerated centrifuge, model 2–16 KL, manufactured by Sigma Laborzentrifugen GmbH in Germany. The samples were spun at a rpm per minute for a duration of 15 min while maintaining a constant temperature of 4 °C. Once the centrifugation was complete, the samples were analyzed using HPLC to identify and quantify the individual polyphenols and to calculate the bioaccessibility index of individual polyphenols.

### 2.5. Determination of Bioaccessibility of Polyphenols

The bioaccessibility of individual polyphenol compounds was determined by subjecting the samples to various stages of gastrointestinal digestion. The equation used to carry out this calculation is based on the research of Iqbal et al. [32]:

% Bioaccessibility = concentration of polyphenol at each phase of digestion/concentration of polyphenol in the supernatant before digestion × 100(5)

### 2.6. Statistical Analysis

The data were declared as mean of determinations performed in triplicate. The one-way analysis of variance (ANOVA) followed by Tukey’s test was used to determine if there were any statistically significant differences between the means of independent samples (*p* < 0.05). A two-way ANOVA analysis was conducted, followed by Tukey’s post hoc test to assess the impact of sample type and digestive phase on phenolic concentration and bioaccessibility index. Statistical analysis was performed using Addinsoft statistical software (version 2022.3.1). A principal component analysis (PCA) was performed with Addinsoft statistical software to correlate variables to which parameters differed between selected samples. The other graphs were drawn with GraphPad-Prism software version 9.03 (San Diego, CA, USA).

## 3. Results

### 3.1. Chemical Analysis

#### 3.1.1. Proximate and Mineral Composition of Black Chokeberry Fruits, Leaves, and Pomace

Proximate and mineral composition are presented in Table 2. Based on the proximate composition, the pomace fraction had the highest average value of dry matter and crude fiber compared to the fruits and leaves.

The chokeberry leaves presented significantly higher levels of crude protein, crude fat, and ash than its fruit and pomace. In fact, the crude protein content in chokeberry leaves is approximately twice that of pomace and over six times that of fruit. According to Table 2, chokeberry fruit had the highest percentage of carbohydrates, followed by pomace and leaves. Copper was not detected in any of the analyzed samples, but higher levels of iron, manganese, and zinc were found in the leaves as compared to the pomace and fruit.

#### 3.1.2. Phytochemicals Quantification of Black Chokeberry Fruits, Leaves, and Pomace

The results obtained for phytochemical composition are presented in Table 3. Accordingly, the leaves have the highest mean value of total polyphenol content; compared to the mean value of fruit, it is twice-fold, while the mean value of pomace fraction, in turn, is approximately three times higher. Black chokeberry fruit presented higher polyphenol content compared to pomace. In the same tendency, the leaves contained higher flavonoids content, followed by fruit and pomace. The leaves had the highest level of lutein and zeaxanthin compared to fruit and pomace. Between black chokeberry pomace and fruit, the pomace contained the highest level of lutein and zeaxanthin. Astaxanthin was not detected in the pomace, while leaves contained significantly higher levels than fruit. The results showed that black chokeberry pomace contained a higher concentration of canthaxanthin than fruit and leaves. In the case of tocopherols, leaves contained high levels of α-tocopherols compared to pomace and fruit. Of all samples, the pomace has the highest content of γ-tocopherols, followed by leaves. Delta tocopherols were not detected in the leaves, but in the pomace, it was present in higher amounts than in the fruit.

#### 3.1.3. Fatty Acids Profile of Black Chokeberry Fruits, Leaves and Pomace

The results obtained for fatty acids composition are presented in Table 4. Regarding saturated fatty acids (SFA), the leaves contained higher levels of C10:0 and C14:0 fatty acids compared to pomace and fruit. C12:0 fatty acids were not detected in chokeberry fruit, but in leaves were found in higher amounts compared to pomace.

Regarding MUFA, C15:1, C16:1, and C18:1 FA were more abundant in leaves compared to pomace and fruit. C18:2n6 FA was detected in a higher quantity in fruit compared to pomace and leaves. C20:2n6, C20:3n6, and C20:4n6 FA were detected only in pomace, while C22:4n6 FA was determined only in the leaves. In the case of the n-3 FA group, a significant difference was observed between pomace, fruits, and leaves. The highest quantity of C18:3n3 was found in the leaves, followed by pomace and fruit. The level of C18:3n3 was 12 times higher in the leaves compared to pomace and approximatively 21 times higher compared to the fruits. C18:4n6 was not significantly different between leaves and pomace, but the pomace had a higher content compared to the fruits. The leaves contained a higher content in total SFA, SFA/UFA, PUFA/MUFA, and n-3 PUFA compared to pomace and fruit. MUFA, UFA, n-6 PUFA, n-6/n-3 were higher in the fruits compared to pomace and leaves samples, while the PUFA was higher in pomace compared to fruit and leaves.

#### 3.1.4. Antioxidant Activity of Black Chokeberry Fruits, Leaves, and Pomace

**Figure 2:**
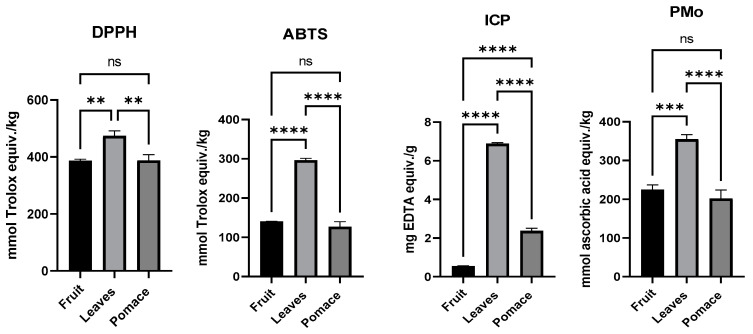
Antioxidant potential of black chokeberry fruits, leaves, and pomace. DPPH—2,2-Diphenyl-1-picrylhydrazyl; ABTS—2,2′-Azinobis-3-Ethylbenzthiazolin-6-Sulfonic Acid; ICP—Iron chelating power; PMo—Phosphomolybdenum method; ns—non significant; ** *p* < 0.01; *** *p* < 0.001; **** *p* < 0.0001.

Figure 2 presents the results of the antioxidant potential tests conducted on the fruit, leaves, and pomace of the black chokeberry. The findings show that the leaves have stronger antioxidant activity, as measured by ABTS^•+^, DPPH^•^, phosphomolybdenum, and iron chelating power methods, compared to the fruit and pomace. There was no noticeable difference in the antioxidant potential between the fruit and the pomace. It is worth noting that the iron chelating power of the pomace was higher than that of the fruit.

### 3.2. Biplot Correlation

**Figure 3:**
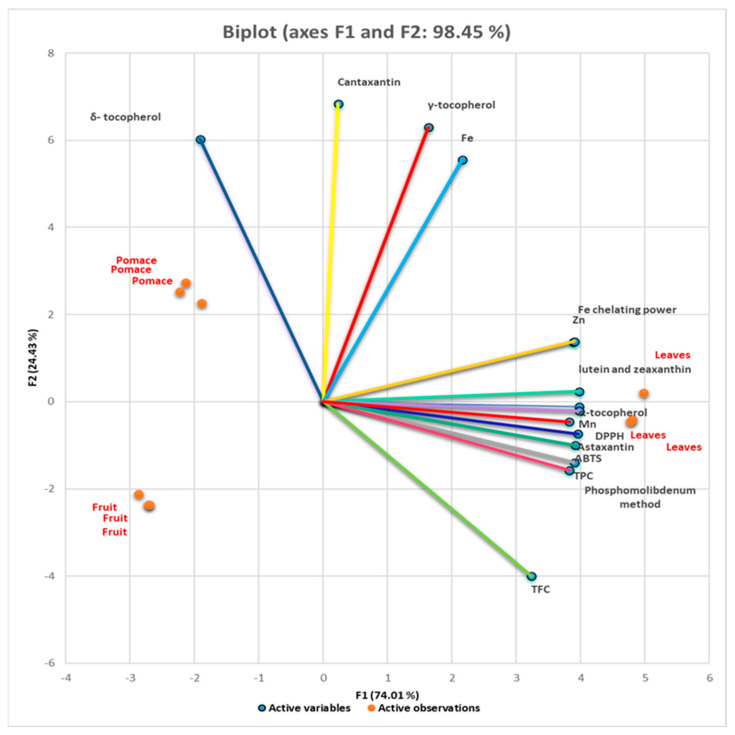
PCA score plot for selected parameters of black chokeberry samples (fruit, leaves, and pomace). TPC—total polyphenol content; TFC—total flavonoid content; ABTS—2,2-Azinobis-3-Ethylbenzthiazolin-6-Sulfonic Acid; DPPH—2,2-Diphenyl-1-picrylhydrazyl. F1 corresponds to PC1, F2 corresponds to PC2.

Figure 3 illustrates the biplot correlation of black chokeberry samples. The results showed that 98.45% of the total variation is explained by the first 2 principal components. PC1 covers 74.01% of the variance, and PC2 covers 24.43% of the variance. According to the biplot correlations, the most important variables in PC1 are the polyphenols, antioxidant activity, α-tocopherol, lutein, and zeaxanthin. In PC2, the most important variables are δ- and γ-tocopherol, canthaxanthin, and Fe. The biplot also discriminates the variables among selected samples. The biplot showed that α-tocopherol, TPC, astaxanthin, Mn, antioxidant activity expressed through ABTS, DPPH, and phosphomolybdenum methods were the most definitory variables for leaves and δ-tocopherol for pomace. According to biplot correlation, the analytical results obtained are not directly related to fruit samples.

### 3.3. In Vitro Gastrointestinal (GI) Digestion of Black Chokeberry Fruits, Leaves, and Pomace

Table 5 displays the polyphenols profile of black chokeberry samples after simulated gastrointestinal digestion. The leaves had the highest content of polyphenols, with chlorogenic acid and epicatechin being the major constituents, followed by fruit and pomace with chlorogenic acid and hydroxybenzoic acid as the main constituents. It is worth noting that protocatechuic acid was exclusively identified in black chokeberry leaves. The present study showed variations in the concentration of phenolic compounds identified in black chokeberry fruit, leaves, and pomace before and during gastrointestinal digestion. The analysis revealed that the concentration of gallic acid was significantly higher in the samples after digestion when compared to the pre-digestion levels. Conversely, the concentration of caffeic acid was observed to be higher in fruit and pomace after intestinal digestion. The most frequently occurring phenolic acid in chokeberry leaves, fruit, and pomace was chlorogenic acid, but its concentration decreased during the digestion process. Overall, the findings suggest that the digestion process affects the concentration of phenolic compounds in black chokeberry samples. The polyphenol content was higher in black chokeberry fruit and pomace (intestinal phase, IP) > leaves (gastric phase, GP). Moreover, the phenolic content varied depending on the specific type of sample and the digestive phase. It can be observed that the content of phenolic acids and flavonoids of both fruit and pomace was significantly higher in the IP > GP > OP (oral phase). During the gastric phase, leaves had a higher level of hydroxybenzoic acids, except gallic acid, than fruits. Gallic acid was higher in leaves (IP) > leaves (GP) > fruit (IP). The black chokeberry fruit contained a higher level of 3-hydroxybenzoic acid (IP) than the fruit (OP) and pomace (IP). Vanillic acid was higher in fruit (IP) > leaves (IP) > pomace (IP), whereas ellagic acid was higher in fruit (IP) than pomace (IP). Among hydroxycinnamic acids, chlorogenic acid was higher in leaves (GP) > leaves (IP) > fruit (IP) > pomace (IP). The cinnamic acid had a higher level in leaves (GP) > fruits (IP) > pomace (IP). Methoxycinnamic acid was higher in leaves (GP) > fruit (IP) > leaves and pomace (IP). Caffeic acid was higher in leaves (IP) > leaves (GP) > fruit (IP) > pomace (IP). Regarding flavonoids class, catechin levels in the digested sample were higher in fruit (GP) > leaves (GP). In addition, epicatechin and epigallocatechin were higher in leaves (GP) > fruit (IP). Quercetin was higher in the fruit (IP) > pomace (IP) > leaves (GP). Rutin was higher in fruit (IP) > leaves (OP). Resveratrol level was higher in fruit (IP) > pomace (IP) > leaves (GP).

According to the data presented in Table 6, gallic acid emerged as the phenolic acid with the highest bioaccessibility index in all black chokeberry samples. Among hydroxycinnamic acids in fruit and pomace, caffeic acid exhibited the highest bioaccessibility index. Notably, the bioaccessibility index for black chokeberry leaves was the highest for cinnamic acid, in addition to gallic acid. The results also indicate that bioaccessibility varied depending on the type of sample and the digestive phase. It can be observed that the bioaccessibility index (BI) of all phenolic acids and flavonoids was significantly higher in IP (intestinal phase) compared to GP (gastric phase) and OP (oral phase) for both fruit and pomace. However, for leaves, except for gallic acid, which has a higher BI in the intestinal phase, all the other phenolic acids and flavonoids (except rutin, which has the highest bioaccessibility in the oral phase) have a significantly higher BI in the GP compared to OP and IP.

Hydroxibenzoic acid (gallic, vanillic, ellagic acid) and hidroxycinnamic acids (ferulic, p-coumaric, caffeic, and methoxycinnamic acids) had higher BI in OP in fruits and pomace compared to leaves. Gallic acid had a higher BI in leaves (IP) > fruit (IP) > pomace (IP). The bioaccessibility index of 3-hydroxybenzoic acid was higher in fruit (IP) > leaves (GP) > pomace (IP). Vanillic acid had a higher BI in fruit (IP) > leaves and pomace (GP) > fruit (GP), whereas ellagic acid had a higher BI in fruit (IP) than pomace (IP).

Among hydroxycinnamic acids, chlorogenic acid had a higher BI in leaves (GP) > leaves (IP) > fruit (IP) > pomace (IP). Interestingly, cinnamic acid had a higher BI in leaves (GP) > fruits (IP) > pomace (IP). Methoxycinnamic acid had a higher BI in leaves (GP) > fruit (IP) > leaves and pomace (IP). The bioaccessibility index of caffeic acid was higher in leaves (IP) > leaves (GP) > fruit (IP) > pomace (IP).

Epicatechin had a higher BI in leaves (IP) > pomace (IP) > leaves (IP). The bioaccessibility index of epigallocatechin was higher in leaves (GP) > fruit (IP). Quercetin had a higher BI in the fruit (IP) > pomace (IP) > leaves (GP). The bioaccessibility index of rutin was higher in fruit (IP) > leaves (OP). Regarding resveratrol, the BI was higher in fruit (GP) > leaves (IP) > leaves (OP).

## 4. Discussion

### 4.1. Proximate and Mineral Composition of Black Chokeberry Fruits, Leaves, and Pomace

Results from the chemical composition of black chokeberry samples indicate that the pomace had a higher concentration of dry matter and crude fiber than fruits and leaves. The observation is due to the constituents of pomace (peels, stones, and internal fruit cell structures), which are a rich source of dietary fiber. Some researchers [3,33,34] found 56 g/kg fresh weight (FW) crude fiber in fruit, 63% to 78% of total fiber content in DM in pomace [10], and 8.98 g/kg crude fiber in DM in leaves [34]. Similar results were declared by Sidor and Gramza-Michałowska [3]. The by-products of black chokeberry, which are rich in dietary fiber, are considered valuable ingredients for food supplements and functional foods [10]. These products can absorb harmful substances such as low-density lipoproteins cholesterol, and bind heavy metals and mineral components, thereby reducing their levels [33]. An adequate intake for total fiber is set at 25–40 g/day [35]. The findings from our study showed that black chokeberry leaves contained significantly higher levels of crude protein (approximately twice compared to pomace and over six times compared to fruit), crude fat, and ash than fruit and pomace. Literature results reported 0.7% FW crude protein in the fruit [33], 10.77% in dried pomace [36], and 11.2% DM in leaves [34]. Studies have shown that the content of crude protein is higher in seeds as compared to other parts of plants [37]. Therefore, it can be inferred that fractions containing more seeds will have a higher content of crude protein. This finding is supported by research conducted by multiple sources [12,36]. In a similar trend, literature reports that the largest amounts of crude fat were found in the pomace, particularly the seed fractions 2.9–13.9% [12]. Contrary to our results, other authors have reported lipid content ranging between 0.09% and 0.17% in fresh chokeberry fruit [33,38], between 3% and 14% on a dry matter basis in pomace [12], and 5.52% on a dry matter basis in leaves [34]. Chemical composition depends on various factors, including climate conditions, soil composition, berry maturity, harvest methods, and storage conditions [3,39].

In this study, black chokeberry leaves had higher levels of Fe, Mn, and Zn compared to pomace and fruit. Our results on leaves and fruit showed higher concentrations of Fe and Mn compared to those reported by Biel et al. [34] and Pavlovic et al. [40]. The concentration of Mn in leaves was found to be over 45 times higher compared to that in fruit and over 13 times higher compared to that in pomace. The concentration of Zn in leaves was 3 times higher compared to that in fruit and approximately 2 times higher compared to that in pomace. In this study, the analyzed pomace contained approximately 15 times more Fe and 2 times more Zn than concentrations reported by Pavlovic et al. [40].

### 4.2. Phytochemicals Quantification of Black Chokeberry Fruits, Leaves and Pomace

Phytochemicals are biofactors that determine the high bioactivity of black chokeberries. The present findings showed differences between the three types of black chokeberry samples. The black chokeberry leaves have a higher content of total polyphenols and flavonoids compared to fruit and pomace fractions. The polyphenol content of leaves was twice as high as in the fruit and approximately three times higher compared to the pomace. Similar results were found by Teleszko et al. [41]. In contrast, it was observed that dried berries exhibited a higher polyphenolic content (1401.32 mg GAE/g) compared to the leaves (765.63 mg GAE/g), surpassing the results obtained in our study [42]. The concentration of phenolic compounds depends on the plant variety, cultivation conditions, and date of harvesting. As reported by Szopa et al. [18], higher total phenolic content (1946 and 9148 mg GAE /100 g) was determined in samples of leaves harvested at a more mature stage. These findings demonstrate that leaves can serve as a valuable source of polyphenols, with potential applications in various food and pharmaceutical fields. In this study, the total polyphenols and flavonoids were found in a higher concentration in black chokeberry fruit compared to pomace. However, it was reported that chokeberry pomace contained the highest total phenolics content, whereas the highest total flavonoid content was in dried fruits [43]. In a study conducted by Petrov Ivanković et al. [44] comparing the total phenolic content of four types of pomaces (chokeberry, blackcurrant, raspberry, and strawberry pomace), it was reported that the highest total phenolic content was found in the chokeberry and blackcurrant pomace extracts. Raspberry and strawberry pomace extract had approximately two times lower total phenolic content than blackcurrant and chokeberry. Other authors [45] analyzing various berry pomaces reported that cranberry, lingonberry, sea buckthorn, and black currant pomace had lower polyphenol content (ranging from 3.89 to 11.06 GAE/g DM) compared to the chokeberry pomace analyzed in our study (22.94 mg GAE/g DM). These findings revealed the valuable phenolic composition of chokeberry pomace compared to other berry pomaces, which may be a functional ingredient source of antioxidant compounds.

In addition to its valuable phenolic content, the results indicate that the leaves and pomace fraction may represent an important source of lipophilic antioxidants such as lutein and zeaxanthin and tocopherols, found in a higher concentration than in the fruit. Lipophilic antioxidants are crucial for human health because of their antioxidant, antiaging, anti-inflammatory, anticancer, and cardioprotective properties [46]. Tocopherols are a group of fat-soluble compounds that are present in all parts of the plant, which function as antioxidants and protect against oxidative stress and lipid peroxidation [47]. In this study, the pomace contained a higher level of tocopherols than fruit. This may be due to the abundance of tocopherols in the seeds, which are retained in the pomace during the pressing process, along with the cell wall compounds containing antioxidant fractions [48].

Fatty acids fulfill essential biological, structural, and functional roles in the human body. The fatty acid composition of the plants, fruit, or by-products is crucial for assessing their nutritional value. Analysis of the composition of higher fatty acids in fruit, pomace, and leaves of black chokeberry showed that the tested samples are a good source of essential fatty acids. In this study, black chokeberry fruit and pomace contained high concentrations of unsaturated, monounsaturated, and polyunsaturated fatty acids. However, the chokeberry pomace was richer in n-3 PUFA compared to the fruits and leaves. Similar results were obtained by [36] when analyzing dried pomace of black chokeberry (73.6% PUFA of total fatty acids), reporting linoleic acid as the main fatty acid. In contrast, others [3] reported that chokeberry pomace contained high levels of oleic acid (C18:1). There was no extended literature on the comparative fatty acid composition of selected black chokeberry samples; most studies focused on phenolic composition. The present study shows for the first time that chokeberry leaves have the highest level of α-linolenic acid (α-C18:3), followed by pomace and fruit (29.28%, 2.43%, and 1.40%, respectively) and the lowest n-6 to n-3 PUFA ratio (1.02 compared to 18.47 in pomace, and 31.36 in fruit). The n-3 and n-6 fatty acids are crucial to cell membranes, however, they cannot be converted in the human body. A lower ratio between those indices is desirable because it exerts suppressive effects on the pathogenesis of many diseases, including cardiovascular disease, cancer, and inflammatory and autoimmune diseases [49].

### 4.3. Antioxidant Potential of Black Chokeberry Berries, Fruits, Leaves, and Pomace

The regular intake of fruits and by-products that are rich in antioxidants has been widely associated with a notable enhancement in overall health and a lower incidence of chronic diseases. Some authors confirmed the antioxidant properties of black chokeberries in various radical scavenging assays and the effects of transition metals on oxidation [34].

The results of the study showed that leaves had more effective free radical scavenging ability (DPPH^•^, ABTS^•+^) and iron chelating power than fruit and pomace. The ABTS^•+^ and DPPH^•^ methods are based on electron transfer assays [50], while iron chelating power showed the ability of antioxidants to bind Fe due to their functional groups that perform metal binding [51]. The methods used encompass different aspects of antioxidant action, providing a broader perspective on the antioxidant potential of aronia. The present study elucidates that, although the black chokeberry leaves are a neglected constituent of the plant (considered useless), they exhibit a significant potential to serve as a source of antioxidants, which can effectively promote a healthy lifestyle. In addition, following a similar approach, other fruit leaves could be explored as novel sources of antioxidants. This highlights the potential for expanding the range of antioxidant sources beyond the traditional ones. Another study [41] revealed that fruit possessed stronger antioxidant activity estimated based on ABTS^•+^ analysis than leaves. The differences could consist in the maturity stage of the plant, development and ripening. In this regard, it was reported that old leaves possess a lower antioxidant activity than young ones [42], and unripe chokeberries exhibited the highest antioxidant activity due to their abundant flavonoid and procyanidin content [52].

The products and post-production waste of chokeberry fruit also have antioxidative potential, in addition to the fruit itself. In this study, black chokeberry pomace possesses higher iron chelating ability than fruit. Similar results were reported by other researchers [53]. When analyzing three types of products (fruit, juice, and pomace), some researchers [3] observed that the pomace had higher antiradical activity against ABTS^•+^ and DPPH^•^ than fruit and juice. On the other hand, a study that also examined the scavenging activity of ABTS^•+^ and DPPH^•^ observed that the chokeberry pomace had higher antioxidant activity than strawberry, raspberry, and blackcurrant pomace [54]. The present accomplishment holds significant value in the actual context as a large amount of pomace is produced during the processing of berry fruits, which is subsequently discarded as waste, leading to a loss of natural antioxidants.

### 4.4. Biplot Correlation from Principal Component Analysis (PCA) of Samples

The PCA showed the differentiation among the black chokeberry samples using a small number of linear combinations of the variables that contribute most significantly to the observed variability in the data. The polyphenols, antioxidant activity, α-tocopherol, lutein and zeaxanthin, astaxanthin, Mn, and Zn had a high loading factor (0.97–0.999) which corresponded to factor 1 of PCA. The δ- and γ-tocopherol, canthaxanthin, and Fe were characterized by factor loads that linked them with the negative part of factor 2 of PCA. Quite interesting is that polyphenols, α-tocopherol, lutein and zeaxanthin, astaxanthin, Mn, and Zn contribute significantly towards the antioxidant activity of leaves, while δ- and γ-tocopherol, canthaxanthin, and Fe, of pomace. Principal cluster analysis showed that the bioactive compounds determined in this study were found to contribute significantly to the antioxidant activity, being positively correlated with the antioxidant activity expressed through the four analytical methods.

### 4.5. In Vitro Gastrointestinal (GI) Digestion

Simulated in vitro gastrointestinal digestion of polyphenols provides information about the transformations that occur during the digestion process to better the bioavailability of polyphenols. Polyphenols possess many pharmacological properties [55]. The outcomes of the static simulated gastrointestinal digestion test showed that the digestion process affects the polyphenol content in black chokeberry’s fruit, leaves, and pomace to different extents. To the best of our knowledge, prior research has not explored the comparative bioaccessibility and recovery of polyphenolic compounds from different black chokeberry samples, including fruit, leaves, and pomace.

The study found that the highest bioaccessibility index in the intestinal phase was recorded for polyphenols identified in black chokeberry fruit and pomace, while for leaves, the highest bioaccessibility index of the majority of polyphenols, except for rutin and gallic acid, was observed in the gastric phase. Studies reported a high bioaccessibility for phenolic compounds of fruits in the intestinal phase [56,57], whereas some authors showed that the bioaccessibility index was higher in the gastric phase because the acidic conditions of the stomach allow higher stability of phenolic compounds [58,59]. The effect of gastrointestinal digestion on bioaccessibility may depend on the nature of the sample, the chemical class of the phenolic compounds, and interactions between polyphenols and other bioactive compounds from the plant matrix that may react through antagonistic or synergistic forms [60].

The results of our study showed that the black chokeberry samples analyzed had a higher concentration of gallic acid after undergoing the process of digestion and, in turn, a higher bioaccessibility index. This increase was significant when compared to the levels of gallic acid in the samples before digestion. The explanation may consist in the catabolism of many phenolic acids which may result in an increase in free phenolic acids in the digestive matrix. Numerous studies have found that the levels of gallic and ellagic acids tend to increase after the process of gastrointestinal digestion. This increase can be attributed to the hydrolysis of gallotannins and ellagitannins, respectively [56,61]. In line with our study, some authors [25] demonstrated through simulated in vitro digestion that hydroxybenzoic acids of raspberry and blackberry leaves had the highest bioaccessibility index in the intestinal phase. Gallic acid was found to be one of the most bioaccessible phenolics. However, the concentration of phenols in plasma can increase based on the presence of metabolites produced in the organism’s tissue or the colon’s microflora [55]. The absorption rate and metabolites in the bloodstream of a polyphenol depend on its chemical composition [62]. The present findings accentuate the potential of black chokeberry as an abundant source of bioactive compounds, particularly bioaccessible phenolic acids. The identified phenolic acids demonstrate the plausible health benefits of black chokeberry, which could be attributed to their antioxidative, anti-inflammatory, and anticancer properties. Hence, black chokeberry could be considered an efficient natural resource for developing functional foods, nutraceuticals, and pharmaceuticals.

In this study, it was found that ellagic acid is vulnerable to hydrolysis in both oral and gastric digestion conditions. In fruit and pomace, its content decreased significantly during these stages (BI of 26.09% in OP, 20.38% in GP for fruit, and BI of 25.85% in OP, 6.92% in GP for pomace), but it increased during the intestinal phase (BI of 69.28% and 56.69%, respectively). This could be due to the association of ellagic acid with intestinal enzymes or dietary fiber carbohydrates from the food matrix. Other authors have reported similar results [58,63]. It was shown that this association is predicted to increase with the molecular weight and the number of hydroxyl groups of phenolics, which may explain the low bioaccessibility of ellagitannins from Jaboticaba peel powder [58]. The increase in bioaccessibility in the IP of gallic acid and ellagic acid indicates that the physiological conditions used in this study can release these phenolic acids as major hydrolytic products from gallotanins and ellagitannins, respectively [63]. Chlorogenic acid is the most abundant phenolic acid within chokeberry leaves, fruit, and pomace. Other researchers [15,64] have also confirmed that chlorogenic acid is the dominant compound among the phenolics present in chokeberry leaves. They observed that this compound is more abundant in the younger leaves collected when they were two months old or even younger. They have noticed that chlorogenic acid content is higher in the younger leaves collected when they were approximately two months old or even younger. However, it is worth noting that in our study, its concentration undergoes a significant decrease during the process of digestion. This observation can be due to the conversion into other chlorogenic acids, such as caffeic acid, by specific esterase by the gut microbiota, as the literature reported [65,66]. Chlorogenic acids, a type of hydroxycinnamic acid derivatives, are naturally esterified. As a result, their absorption is impeded [67]. Moreover, during digestion, the polyphenols found in black chokeberry samples exhibit distinct behaviors. After oral digestion, the recovery of chlorogenic acid decreased in fruit and pomace samples (10.13% and 5.50% bioaccessibility) but increased eight times in the leaves (37.08% bioaccessibility). After gastric digestion, in the intestinal phase, the same predominant phenolic acid and chlorogenic acid increased in fruit and pomace and decreased in leaves. This different behavior could be explained by the nature of the sample and the interactions with other components in the matrix. For example, the black chokeberry leaves have a high content of fiber, which, in the gastric phase under the action of gastric enzymes, interacts with polyphenols and decreases their absorption and, in turn, their bioaccessibility. Recent studies have reported that dietary fibers are the primary carriers of phenolic compounds, and their interaction may hinder polyphenols’ bioaccessibility by providing a physical barrier against acidic gastric conditions [68,69]. According to some authors [70], the complex structure of a plant’s cell wall leads to a low bioaccessibility of a significant percentage of hydroxycinnamic acids. On the other hand, some authors [71] have suggested that phenolic compounds can bind to dietary components like minerals and proteins, which can protect against degradation processes.

Among the various hydroxycinnamic acids, caffeic acid exhibited the highest bioaccessibility index in the fruit and pomace samples. In contrast, black chokeberry leaves showed a higher bioaccessibility index for cinnamic acid. Hydroxycinnamic acids have potent antioxidant properties that prevent diseases associated with oxidative stress, such as cardiovascular and neurodegenerative diseases and cancer [72]. Additionally, several derivatives possess anti-inflammatory and antimicrobial activities [73]. The outcomes of this study bear significant implications for the utilization of these compounds in food and nutraceutical products, as well as in the pharmaceutical industry.

Quercetin has been identified as the flavonol with the highest bioaccessibility in fruit and pomace. The high bioaccessibility after digestion of black chokeberry fruit (BI of 98.72%) and pomace (BI of 64.69%) indicates no degradation of the quercetin. In leaves, its intestinal bioaccessibility was similar to that in the oral phase. A high stability in the stomach environment of quercetin was observed for fruit and leaves. Flavonoids found in fruits and vegetables are predominantly present in a glycosylated form. This implies that they are linked to carbohydrates such as glucose, maltose, and others, thereby rendering them more stable under gastrointestinal conditions [74].

## 5. Conclusions

The study compares the nutritional content of different parts of black chokeberry (fruit, leaves, and pomace) and highlights the nutritional value of by-products. The results demonstrated that leaves had the higher concentrations of biologically active substances (total polyphenols—61.06 mg GAE/g, total flavonoids—8.47 mg QE/g, total xanthophylls—2353.24 mg/kg, and total tocopherols—1172.2 mg/kg) and superior in vitro antioxidant activity. The phenolic content of black chokeberry samples significantly changed in the different digestion stages. The highest bioaccessibility index in the intestinal phase was recorded for polyphenols identified in black chokeberry fruit and pomace. In conclusion, residues from black chokeberry fruit processing, such as leaves and pomace, are also valuable sources for the recovery of bioactive compounds, but the pomace has a higher polyphenol bioaccessibility than leaves and can be a promising strategy for developing antioxidant-based dietary supplements that can cater to the growing demand for healthy products concomitant with the circular economy.

## Figures and Tables

**Table 1:** Gradient evolution data for determination of polyphenols.

Time (min)	Solvent A (%)	Solvent B (%)	Solvent C (%)
0–15	90	5	5
15–20	81	4	15
20–25	72	3	25
25–40	60	2	38
40–50	90	5	5

**Table 2:** Proximate and mineral composition determined in the black chokeberry fruits, leaves, and pomace.

Analyzed Parameters	Fruit	Leaves	Pomace	SEM	*p*-Value
Proximate composition (%)					
Dry matter	91.76 ^b^	90.92 ^c^	94.9 ^a^	0.004	<0.0001
Crude protein	1.53 ^c^	10.11 ^a^	5.25 ^b^	0.003	<0.0001
Crude fat	4.17 ^b^	6.75 ^a^	2.51 ^c^	0.001	<0.0001
Crude fiber	8.29 ^c^	13.33 ^b^	14.3 ^a^	0.004	<0.0001
Ash	2.01 ^c^	7.82 ^a^	2.41 ^b^	0.004	<0.0001
Carbohydrates	75.78 ^a^	52.93 ^c^	70.44 ^b^	0.002	<0.0001
Mineral composition (mg/kg)					
Copper	nd	nd	nd	-	-
Iron	72.93 ^b^	94.29 ^a^	94.27 ^a^	2.499	0.001
Manganese	4.54 ^c^	205.48 ^a^	15.3 ^b^	1.834	<0.0001
Zinc	6.67 ^c^	20.13 ^a^	10.54 ^b^	0.356	<0.0001

Different letters (^a,b,c^) in the same row show significant statistical differences according to ANOVA test (*p* < 0.05); nd—not detected; SEM—standard error of the mean.

**Table 3:** Phytochemicals quantification in the black chokeberry fruit, leaves, and pomace.

Nutrient *	Fruit	Leaves	Pomace	SEM	*p*-Value
**Polyphenols**					
Total polyphenol content, mg GAE/g	27.99 ^b^	61.06 ^a^	22.94 ^c^	0.724	<0.0001
Total flavonoid content, mg QE/g	5.23 ^b^	8.47 ^a^	1.89 ^c^	0.262	<0.0001
**Xanthophylls**					
Lutein and zeaxanthin, mg/kg	70.34 ^c^	2245.99 ^a^	339.22 ^b^	0.005	<0.0001
Astaxanthin, mg/kg	2.41 ^b^	100.29 ^a^	nd	0.013	<0.0001
Canthaxanthin, mg/kg	2.22 ^c^	6.96 ^b^	11.99 ^a^	0.092	<0.0001
**Total xanthophylls**	75.45 ^c^	2353.24 ^a^	351.21 ^b^	0.004	<0.0001
**Tocopherols**					
α-tocopherol, mg/kg	33.46 ^c^	1154.10 ^a^	114.37 ^b^	0.383	<0.0001
γ-tocopherol, mg/kg	5.01 ^c^	18.10 ^b^	21.19 ^a^	0.015	<0.0001
δ-tocopherol, mg/kg	2.13 ^b^	nd	21.63 ^a^	0.028	<0.0001
**Total tocopherols, mg/kg**	38.48 ^c^	1172.20 ^a^	157.19 ^b^	0.359	<0.0001

Different letters (^a,b,c^) in the same row show significant statistical differences according to the ANOVA test (*p* < 0.05); * results expressed to dry matter. GAE—gallic acid equivalents; QE—quercetin equivalents; nd—not detected; SEM—standard error of the mean.

**Table 4:** Fatty acid profile determined in the black chokeberry fruit, leaves, and pomace.

Fatty Acids (%)	Fruit	Leaves	Pomace	SEM	*p*-Value
**Σ Saturated fatty acids**					
C10:0	0.128 ^c^	0.634 ^a^	0.312 ^b^	0.004	<0.0001
C12:0	nd	1.197 ^a^	0.086 ^b^	0.004	<0.0001
C14:0	0.238 ^c^	1.567 ^a^	0.356 ^b^	0.002	<0.0001
C15:0	nd	nd	0.236	-	-
C16:0	8.574 ^c^	22.366 ^a^	8.945 ^b^	0.042	<0.0001
C17:0	nd	0.533 ^a^	0.165 ^b^	0.020	<0.0001
C18:0	1.723 ^c^	3.904 ^a^	1.994 ^b^	0.046	<0.0001
**Σ Monounsaturated fatty acids**					
C15:1	0.117 ^b^	0.854 ^a^	0.058 ^c^	0.010	<0.0001
C16:1	0.240 ^b^	1.444 ^a^	0.281 ^b^	0.015	<0.0001
C17:1	nd	nd	0.168	-	-
C18:1	22.844 ^a^	6.883 ^c^	19.709 ^b^	0.003	<0.0001
**Σ n-6**					
C18:2n6	64.090 ^a^	10.145 ^c^	62.591 ^b^	0.104	<0.0001
C20:2n6	nd	nd	0.268	-	-
C20:3n6	nd	nd	0.262	-	-
C20:4n6	nd	nd	0.260	-	-
C22:2n6	nd	4.885 ^a^	0.310 ^b^	0.001	<0.0001
C22:4n6	nd	15.464	nd	-	-
**Σ n-3**					
C18:3n3	1.408 ^c^	29.284 ^a^	2.434 ^b^	0.014	<0.0001
C18:4n3	0.637 ^b^	0.715 ^ab^	0.766 ^a^	0.216	0.009
C20:3n3	nd	nd	0.252	-	-
Other fatty acids	nd	0.147 ^b^	0.548 ^a^	0.018	<0.0001
**Nutritional quality indices of the lipids**					
Σ SFA	10.780 ^c^	30.205 ^a^	12.097 ^b^	0.002	<0.0001
Σ MUFA	23.085 ^a^	9.165 ^c^	20.217 ^b^	0.003	<0.0001
Σ PUFA	66.135 ^b^	60.494 ^c^	67.140 ^a^	0.009	<0.0001
Σ UFA	89.220 ^a^	69.654 ^c^	87.357 ^b^	0.001	<0.0001
SFA/UFA	0.121 ^c^	0.427 ^a^	0.139 ^b^	0.011	<0.0001
PUFA/MUFA	2.865 ^c^	6.597 ^a^	3.321 ^b^	0.006	<0.0001
n-3	2.045 ^c^	29.994 ^a^	3.450 ^b^	0.032	<0.0001
n-6	64.090 ^a^	30.505 ^c^	63.690 ^b^	0.071	<0.0001
n-6/n-3	31.359 ^a^	1.024 ^c^	18.467 ^b^	0.343	<0.0001

SFA—saturated fatty acids; UFA—total unsaturated fatty acids; MUFA—monounsaturated fatty acids; PUFA—polyunsaturated fatty acids; Different letters (^a,b,c^) in the same row show significant statistical differences according to ANOVA test (*p* < 0.05); nd—not detected; SEM—standard error of the mean.

**Table 5:** Concentrations of polyphenols of undigested chokeberry samples (fruit, leaves, and pomace) and released upon simulated gastrointestinal digestion.

Compound	Fruits				Leaves				Pomace				*p*-Values		
	Undigested	OP	GP	IP	Undigested	OP	GP	IP	Undigested	OP	GP	IP	Type of Sample	Digestive Phase	Type * Digestive Phase
**Phenolic acids**															
**Hydroxybenzoic acids**															
Syringic acid	0.039 ^a^	0.012 ^d^	0.012 ^d^	0.018 ^c^	0.022 ^b^	0.006 ^f^	0.012 ^d^	0.008 ^e^	nd	nd	nd	nd	<0.0001	<0.0001	<0.0001
Gallic acid	0.032 ^f^	0.006 ^h^	0.019 ^g^	0.090 ^d^	0.212 ^b^	0.032 ^f^	0.105 ^c^	0.712 ^a^	0.062 ^e^	0.016 ^g^	0.006 ^h^	0.102 ^c^	<0.0001	<0.0001	<0.0001
3- Hydroxybenzoic acid	0.717 ^a^	0.191 ^e^	0.185 ^ef^	0.447 ^c^	0.403 ^d^	0.043 ^h^	0.165 ^ef^	0.054 ^h^	0.541 ^b^	0.098 ^g^	0.030 ^h^	0.159 ^f^	<0.0001	<0.0001	<0.0001
Vanillic acid	0.224 ^c^	0.056 ^h^	0.049 ^h^	0.126 ^e^	0.668 ^a^	0.096 ^f^	0.300 ^b^	0.102 ^f^	0.159 ^d^	0.040 ^i^	0.011 ^j^	0.075 ^g^	<0.0001	<0.0001	<0.0001
Ellagic acid	0.129 ^a^	0.034 ^e^	0.026 ^f^	0.089 ^b^	nd	nd	nd	nd	0.085 ^c^	0.022 ^g^	0.006 ^h^	0.048 ^d^	<0.0001	<0.0001	<0.0001
Protocatechuic acid	nd	nd	nd	nd	0.089 ^b^	0.028 ^d^	0.142 ^a^	0.042 ^c^	nd	nd	nd	nd	-	<0.0001	-
**Hydroxycinnamic acids**															
p-Coumaric acid	0.108 ^c^	0.017 ^f^	0.011 ^g^	0.041 ^e^	0.973 ^a^	0.021 ^f^	0.324 ^b^	0.064 ^d^	0.019 ^f^	0.001 ^h^	nd	0.003 ^h^	<0.0001	<0.0001	<0.0001
Caffeic acid	0.036 ^g^	0.023 ^h^	0.023 ^h^	0.093 ^d^	0.222 ^a^	0.037 ^fg^	0.120 ^c^	0.144 ^b^	0.041 ^f^	0.014 ^i^	0.003 ^j^	0.079 ^e^	<0.0001	<0.0001	<0.0001
Ferulic acid	0.161 ^c^	0.035 ^g^	0.031 ^g^	0.094 ^e^	0.694 ^a^	0.076 ^f^	0.351 ^b^	0.100 ^e^	0.108 ^d^	0.030 ^g^	0.005 ^h^	0.078 ^f^	<0.0001	<0.0001	<0.0001
Chlorogenic acid	2.713 ^c^	0.353 ^h^	0.278 ^i^	0.783 ^f^	17.954 ^a^	0.767 ^f^	6.657 ^b^	1.581 ^d^	1.415 ^e^	0.329 ^h^	0.071 ^j^	0.469 ^g^	<0.0001	<0.0001	<0.0001
Cinnamic acid	0.006 ^b^	0.001 ^h^	nd	0.004 ^c^	0.004 ^c^	0.001 ^g^	0.004 ^d^	0.003 ^e^	0.009 ^a^	0.001 ^i^	nd	0.003 ^f^	<0.0001	<0.0001	<0.0001
Methoxycinnamic acid	0.329 ^b^	0.054 ^f^	0.030 ^g^	0.158 ^d^	0.835 ^a^	0.031 ^g^	0.310 ^c^	0.058 ^f^	0.144 ^e^	0.019 ^h^	0.003 ^i^	0.059 ^f^	<0.0001	<0.0001	<0.0001
**Flavonoids**															
**Flavanols**															
Catechin	0.025 ^b^	nd	0.065 ^f^	nd	0.088 ^a^	0.006 ^d^	0.018 ^c^	0.003 ^e^	-	nd	nd	nd	<0.0001	<0.0001	<0.0001
Epicatechin	0.546 ^c^	0.118 ^i^	0.063 ^k^	0.266 ^g^	4.250 ^a^	0.288 ^f^	1.789 ^b^	0.426 ^d^	0.408 ^e^	0.090 ^j^	0.020 ^l^	0.164 ^h^	<0.0001	<0.0001	<0.0001
Epigallocatechin	0.141 ^b^	0.021 ^g^	0.032 ^ef^	0.046 ^d^	0.221 ^a^	0.032 ^e^	0.115 ^c^	0.030 ^f^	nd	nd	nd	nd	<0.0001	<0.0001	<0.0001
**Flavonols**															
Quercetin	0.007 ^a^	0.001 ^de^	0.002 ^d^	0.007 ^a^	0.004 ^c^	0.001 ^f^	0.002 ^e^	0.001 ^f^	0.006 ^b^	0.001 ^f^	nd	0.004 ^c^	<0.0001	<0.0001	<0.0001
Rutin	0.153 ^a^	0.022 ^d^	0.015 ^f^	0.076 ^b^	0.048 ^c^	0.019 ^de^	0.008 ^g^	0.016 ^ef^	nd	nd	nd	nd	<0.0001	<0.0001	<0.0001
**Stilbene**															
Resveratrol	0.006 ^c^	0.001 ^i^	0.001 ^h^	0.002 ^f^	0.006 ^b^	0.001 ^i^	0.004 ^e^	0.001 ^hi^	0.008 ^a^	0.002 ^g^	nd	0.005 ^d^	<0.0001	<0.0001	<0.0001

Polyphenols were detected by HPLC and values are expressed in mg/g (dw); OP, Oral phase; GP, Gastric phase; IP, Intestinal phase. Different lowercase superscripts (^a–l^) in each row indicate the interactions between the type of sample and digestive phase; nd—not detected.

**Table 6:** Bioaccessibility index (%) of digested black chokeberry samples.

Compound	Fruit			Leaves			Pomace			*p*-Values		
	Bioaccessibility Index (%)									Type of Sample	Digestive Phase	Type * Digestive Phase
	OP	GP	IP	OP	GP	IP	OP	GP	IP			
**Phenolic acids**												
**Hydroxybenzoic acids**												
Syringic acid	29.38 ^d^	29.69 ^d^	44.61 ^b^	27.08 ^d^	54.29 ^a^	35.14 ^c^	-	-	-	<0.0001	<0.0001	<0.0001
Gallic acid	17.69 ^fg^	58.16 ^d^	290.76 ^b^	15.34 ^gh^	49.54 ^e^	336.32 ^a^	24.59 ^f^	10.10 ^h^	158.61 ^c^	<0.0001	<0.0001	<0.0001
3- Hydroxybenzoic acid	29.19 ^c^	24.81 ^c^	66.40 ^a^	10.64 ^e^	40.86 ^b^	13.37 ^de^	18.96 ^d^	6.51 ^f^	27.47 ^c^	<0.0001	<0.0001	<0.0001
Vanillic acid	27.42 ^c^	21.52 ^c^	58.28 ^a^	14.33 ^d^	44.96 ^b^	15.26 ^d^	25.36 ^c^	7.48 ^e^	43.71 b	<0.0001	<0.0001	<0.0001
Ellagic acid	26.09 ^c^	20.38 ^d^	69.28 ^a^	-	-	-	25.85 ^c^	6.92 ^e^	56.69 ^b^	<0.0001	<0.0001	<0.0001
Protocatechuic acid	-	-	-	31.68 ^c^	160.80 ^a^	46.92 ^b^	-	-	-	-	<0.0001	-
**Hydroxycinnamic acids**												
p-Coumaric acid	18.86 ^c^	9.53 ^e^	42.07 ^a^	2.11 ^gh^	33.28 ^b^	6.62 ^ef^	4.83 ^fg^	1.22 ^h^	13.97 ^d^	<0.0001	<0.0001	<0.0001
Caffeic acid	66.26 ^c^	58.85 ^c^	268.27 ^a^	16.57 ^e^	54.11 ^c^	65.06 ^c^	32.45 ^d^	8.77 ^e^	188.49 ^b^	<0.0001	<0.0001	<0.0001
Ferulic acid	23.71 ^de^	18.86 ^ef^	60.33 ^b^	10.87 ^g^	50.57 ^c^	14.41 ^fg^	29.69 ^d^	3.59 ^h^	66.80 ^a^	<0.0001	<0.0001	<0.0001
Chlorogenic acid	13.80 ^e^	10.13 ^f^	28.84 ^c^	4.27 ^h^	37.08 ^a^	8.81 ^g^	23.30 ^d^	5.50 ^h^	31.90 ^b^	<0.0001	<0.0001	<0.0001
Cinnamic acid	20.99 ^f^	9.20 ^g^	74.43 ^b^	33.49 ^d^	90.92 ^a^	69.83 ^c^	7.39 ^g^	1.20 ^h^	27.39 ^e^	<0.0001	<0.0001	<0.0001
Methoxycinnamic acid	18.19 ^d^	8.69 ^f^	49.87 ^a^	3.67 ^g^	37.11 ^c^	6.94 ^f^	13.26 ^e^	2.45 ^g^	38.15 ^b^	<0.0001	<0.0001	<0.0001
**Flavonoids**												
**Flavanols**												
Catechin	-	-	-	7.32 ^b^	20.63 ^a^	2.96 ^c^	-	-	-	-	0.039	-
Epicatechin	23.13 ^c^	11.33 ^d^	48.34 ^a^	6.77 ^e^	42.09 ^b^	10.03 ^d^	22.26 ^c^	5.34 ^e^	37.02 ^b^	<0.0001	<0.0001	<0.0001
Epigallocatechin	14.76 ^d^	21.21 ^c^	32.63 ^b^	14.63 ^d^	52.18 ^a^	13.39 ^d^	-	-	-	<0.0001	<0.0001	<0.0001
**Flavonols**												
Quercetin	26.80 ^d^	28.29 ^d^	98.72 ^a^	19.21 ^e^	35.63 ^c^	19.69 ^e^	16.25 ^e^	4.89 ^f^	64.69 ^b^	<0.0001	<0.0001	<0.0001
Rutin	15.08 ^d^	9.49 ^e^	49.61 ^a^	39.19 ^b^	16.50 ^d^	33.96 ^c^	-	-	-	<0.0001	<0.0001	<0.0001
**Stilbene**												
Resveratrol	20.89 ^c^	14.93 ^e^	45.16 ^b^	12.63 ^e^	60.05 ^a^	15.51 ^de^	20.91 ^cd^	3.97 ^f^	55.23 ^a^	<0.0001	<0.0001	0.0001

OP—Oral phase; GP—Gastric phase; IP—Intestinal phase. Different lowercase superscripts (^a–h^) in each row indicate the interactions between the type of sample and digestive phase.

## Data Availability

The original contributions presented in the study are included in the article, further inquiries can be directed to the corresponding author.

## References

[1] Martins M.S., Gonçalves A.C., Alves G., Silva L.R. (2023). Blackberries and Mulberries: Berries with Significant Health-Promoting Properties. Int. J. Mol. Sci..

[2] Nile S.H., Park S.W. (2014). Edible berries: Bioactive components and their effect on human health. Nutrition.

[3] Sidor A., Gramza-Michałowska A. (2019). Black Chokeberry *Aronia melanocarpa* L.—A qualitative composition, phenolic profile and antioxidant potential. Molecules.

[4] Cvetanović A., Zenginb G., Zekovića Z., Švarc-Gajića J., Ražić S., Damjanović A., Mašković P., Mitić M. (2018). Comparative in vitro studies of the biological potential and chemical composition of stems, leaves and berries Aronia melanocarpa’s extracts obtained by subcritical water extraction. Food Chem. Toxicol..

[5] Staszowska-Karkut M., Materska M. (2020). phenolic composition, mineral content, and beneficial bioactivities of leaf extracts from black currant (*Ribes nigrum* L.), raspberry (*Rubus idaeus*), and aronia (*Aronia melanocarpa*). Nutrients.

[6] Buda V., Andor M., Diana A., Ardelean F., Pavel I.Z., Dehelean C., Danciu C. (2020). Cardioprotective effects of cultivated black chokeberries (*Aronia* spp.): Traditional uses, phytochemistry and therapeutic effects. Bioactive Compounds in Nutraceutical and Functional Food for Good Human Health.

[7] Oszmiański J., Wojdyło A. (2005). Aronia melanocarpaphenolics and their antioxidant activity. Eur. Food Res. Technol..

[8] Raczkowska E., Nowicka P., Wojdyło A., Styczyńska M., Lazar Z. (2022). Chokeberry pomace as a component shaping the content of bioactive compounds and nutritional, health-promoting (anti-diabetic and antioxidant) and sensory properties of shortcrust pastries sweetened with sucrose and erythritol. Antioxidants.

[9] Sarv V., Venskutonis P.R., Rätsep R., Aluvee A., Kazernavičiūtė R., Bhat R. (2021). Antioxidants characterization of the fruit, juice, and pomace of sweet rowanberry (*Sorbus aucuparia* L.) cultivated in Estonia. Antioxidants.

[10] Jurendi’c T., Ščetar M. (2021). Aronia melanocarpa products and by-products for health and nutrition: A Review. Antioxidants.

[11] Mayer-Miebach E., Adamiuk M., Behsnilian D. (2012). Stability of chokeberry bioactive polyphenols during juice processing and stabilization of a polyphenol-rich material from the by-product. Agriculture.

[12] Sójka M., Kołodziejczyk K., Milala J. (2013). Polyphenolic and basic chemical composition of black chokeberry industrial by-products. Ind. Crops Prod..

[13] Negreanu-Pirjol B.S., Oprea O.C., Negreanu-Pirjol T., Roncea F.N., Prelipcean A.M., Craciunescu O., Iosageanu A., Artem V., Ranca A., Motelica L. (2023). Health Benefits of antioxidant bioactive compounds in the fruits and leaves of *Lonicera caerulea* L. and *Aronia melanocarpa* (Michx.) Elliot. Antioxidants.

[14] De Ancos B., Colina-Coca C., González-Peña D., Sánchez-Moreno C., Gupta V.K., Tuohy M.G. (2015). Bioactive compounds from vegetable and fruit by-products. Biotechnology of Bioactive Compounds.

[15] Wojdyło A., Nowicka P. (2021). Profile of Phenolic Compounds of Prunus armeniaca L. leaf extract determined by LC-ESI-QTOF-MS/MS and their antioxidant, anti-diabetic, anti-cholinesterase, and anti-inflammatory potency. Antioxidants.

[16] Untea A.E., Varzaru I., Saracila M., Panaite T.D., Oancea A.G., Vlaicu P.A., Grosu I.A. (2023). Antioxidant properties of cranberry leaves and walnut meal and their effect on nutritional quality and oxidative stability of broiler breast meat. Antioxidants.

[17] Vlaicu P.A., Untea A.E., Varzaru I., Saracila M., Oancea A.G. (2023). Designing nutrition for health—Incorporating dietary by-products into poultry feeds to create functional foods with insights into health benefits, risks, bioactive compounds, food component functionality and safety regulations. Foods.

[18] Szopa A., Kokotkiewicz A., Kubica P., Banaszczak P., Wojtanowska-Krośniak A., Krosniak M., Marzec-Wróblewska U., Badura A., Zagrodzki P., Bucinski A. (2017). Comparative analysis of different groups of phenolic compounds in fruit and leaf extracts of *Aronia* sp.: *A. melanocarpa*, *A. arbutifolia*, and *A. ×prunifolia* and their antioxidant activities. Eur. Food Res. Technol..

[19] AOAC (2000). International A: Official Methods of Analysis of the AOAC International.

[20] Untea A., Criste R.C., Vladescu L. (2012). Development and validation of a microwave digestion–FAAS procedure for Cu, Mn and Zn determination in liver. Rev. Chim..

[21] Turcu R.P., Panaite T.D., Untea A.E., Vlaicu P.A., Badea I.A., Mironeasa S. (2021). Effects of grape seed oil supplementation to broilers diets on growth performance, meat fatty acids, health lipid indices and lipid oxidation parameters. Agriculture.

[22] Untea A., Lupu A., Saracila M., Panaite T. (2018). Comparison of ABTS, DPPH, phosphomolybdenum assays for estimating antioxidant activity and phenolic compounds in five different plant extracts. Bull. Univ. Agric. Sci. Vet. Med. Cluj-Napoca. Anim. Sci. Biotechnol..

[23] Varzaru I., Untea A.E., Van I. (2015). Distribution of nutrients with benefic potential for the eyes in several medicinal plants. Rom. Biotechnol. Lett..

[24] Zou Y., Lu Y., Wei D. (2004). Antioxidant activity of a flavonoid-rich extract of *Hypericum perforatum* L. in vitro. J. Agric. Food Chem..

[25] Varzaru I., Oancea A.G., Vlaicu P.A., Saracila M., Untea A.E. (2023). Exploring the antioxidant potential of blackberry and raspberry leaves: Phytochemical analysis, scavenging activity, and in vitro polyphenol bioaccessibility. Antioxidants.

[26] Qwele K., Hugo A., Oyedemi S.O., Moyo B., Masika P.J., Muchenje V. (2013). Chemical composition, fatty acid content and antioxidant potential of meat from goats supplemented with Moringa (*Moringa oleifera*) leaves, sunflower cake and grass hay. Meat Sci..

[27] Saracila M., Untea A.E., Varzaru I., Panaite T.D., Vlaicu P.A. (2023). Comparative effects on using bilberry leaves in broiler diet reared under thermoneutral conditions vs. heat stress on performance, health status and gut microbiota. Life.

[28] Sabeena Farvin K.H., Andersen L.L., Nielsen H.H., Jacobsen C., Jakobsen G., Johansson I., Jessen F. (2014). Antioxidant activity of Cod (*Gadus morhua*) protein hydrolysates: In vitro assays and evaluation in 5% fish oil-in-water emulsion. Food Chem..

[29] Santos J.S., Brizola V.R.A., Granato D. (2017). High-throughput assay comparison and standardization for metal chelating capacity screening: A proposal and application. Food Chem..

[30] Prieto P., Pineda M., Aguilar M. (1999). Spectrophotometric quantitation of antioxidant capacity through the formation of a phosphomolybdenum complex: Specific application to the determination of vitamin E. Anal. Biochem..

[31] Minekus M., Alminger M., Alvito P., Ballance S., Bohn T.O., Bourlieu C., Carrière F., Boutrou R., Corredig M., Dupont D. (2014). A standardised static in vitro digestion method suitable for food–an international consensus. Food Funct..

[32] Iqbal Y., Ponnampalam E.N., Le H.H., Artaiz O., Muir S.K., Jacobs J.L., Cottrell J.J., Dunshea F.R. (2022). Assessment of Feed Value of Chicory and Lucerne for Poultry, Determination of Bioaccessibility of Their Polyphenols and Their Effects on Caecal Microbiota. Fermentation.

[33] Tanaka T., Tanaka A. (2001). Chemical components and characteristics of black chokeberry. J. Jpn. Soc. Food Sci. Technol..

[34] Biel W., Jaroszewska A. (2017). The nutritional value of leaves of selected berry species. Sci. Agric..

[35] EFSA Panel on Dietetic Products (2010). Scientific opinion on dietary reference values for carbohydrates and dietary fibre. EFSA J..

[36] Pieszka M., Gogol P., Pietras M., Pieszka M. (2015). Valuable components of dried pomaces of chokeberry, black currant, strawberry, apple and carrot as a source of natural antioxidants and nutraceuticals in the animal diet. Ann. Anim. Sci..

[37] Blejan A.M., Nour V., Păcularu–Burada B., Popescu S.M. (2023). Wild bilberry, blackcurrant, and blackberry by–products as a source of nutritional and bioactive compounds. Int. J. Food Prop..

[38] Lancrajan I. (2012). Aronia melanocarpa a potential therapeutic agent. Stud. Univ. Vasile Goldiş Ser. Ştiinţele Vieţii.

[39] Tolić M.-T., Krbavčić I.P., Vujević P., Milinović B., Jurčević I.L., Vahčić N. (2017). Effects of weather conditions on phenolic content and antioxidant capacity in juice of chokeberries (*Aronia Melanocarpa* L.). Pol. J. Food Nutr. Sci..

[40] Pavlović A.N., Brcanović J.M., Veljković J.N., Mitić S.S., Tošić S.B., Kaličanin B.M., Kostić D.A., Dordević M.S., Velimirović D.S. (2015). Characterization of commercially available products of aronia according to their metal content. Fruits.

[41] Teleszko M., Wojdyło A. (2015). Comparison of phenolic compounds and antioxidant potential between selected edible fruits and their leaves. J. Funct. Foods.

[42] Thi N.D., Hwang E.S. (2014). Bioactive compound contents and antioxidant activity in aronia (*Aronia melanocarpa*) leaves collected at different growth stages. Prev. Nutr. Food Sci..

[43] Bahtinur K., Neradová E., Čížková H., Voldřich M., Rajchl A., Capanoglu E. (2013). Investigating the antioxidant potential of chokeberry (*Aronia melanocarpa*) products. J. Food Nutr. Res..

[44] Petrov Ivanković A., Ćorović M., Milivojević A., Simović M., Banjanac K., Veljković M., Bezbradica D. (2024). Berries pomace valorization: From waste to potent antioxidants and emerging skin prebiotics. Int. J. Fruit Sci..

[45] Jurevičiūtė I., Keršienė M., Bašinskienė L., Leskauskaitė D., Jasutienė I. (2022). Characterization of berry pomace powders as dietary fiber-rich food ingredients with functional properties. Foods.

[46] Shin G.H., Kim J.T., Park H.J. (2015). Recent developments in nanoformulations of lipophilic functional foods. Trends Food Sci. Technol..

[47] Hofius D., Sonnewald U. (2003). Vitamin E biosynthesis: Biochemistry meets cell biology. Trends Plant Sci..

[48] Struck S., Plaza M., Turner C., Rohm H. (2016). Berry pomace–a review of processing and chemical analysis of its polyphenols. Int. J. Food Sci. Technol..

[49] Vlaicu P.A., Panaite T.D., Turcu R.P. (2021). Enriching laying hens eggs by feeding diets with different fatty acid composition and antioxidants. Sci. Rep..

[50] Huang D., Ou B., Prior R.L. (2005). The chemistry behind antioxidant capacity assays. J. Agric. Food Chem..

[51] Gulcin İ., Alwasel S.H. (2022). Metal ions, metal chelators and metal chelating assay as antioxidant method. Processes.

[52] Gralec I.W.M. (2019). Aronia Melanocarpa berries: Phenolics composition and antioxidant properties changes during fruit development and ripening. Emir. J. Food Agric..

[53] Sobhy R., Öz F., Lorenzo J.M., Bakry A.M., Mohamed A. (2023). Bioactive components and health promoting effect of berry by-products. Berry Bioactive Compound By-Products.

[54] Pachołek B., Krawczyk K., Żak E. (2014). Potential use of dried fruit pomaces to create sensory properties and antioxidant activity of fruit teas. Towaroznawcze Problemy Jakości. Pol. J. Commod. Sci..

[55] Stromsnes K., Lagzdina R., Olaso-Gonzalez G., Gimeno-Mallench L., Gambini J. (2021). Pharmacological Properties of Polyphenols: Bioavailability, Mechanisms of Action, and Biological Effects in In Vitro Studies, Animal Models, and Humans. Biomedicines.

[56] Chait Y.A., Gunenc A., Bendali F., Hosseinian F. (2020). Simulated gastrointestinal digestion and in vitro colonic fermentation of carob polyphenols: Bioaccessibility and bioactivity. LWT.

[57] de Araújo F.F., de Paulo Farias D., Neri-Numa I.A., Dias-Audibert F.L., Delafiori J., de Souza F.G., Pastore G.M. (2021). Gastrointestinal bioaccessibility and bioactivity of phenolic compounds from araçá-boi fruit. LWT.

[58] Quatrin A., Rampelotto C., Pauletto R., Maurer L.H., Nichelle S.M., Klein B., Emanuelli T. (2020). Bioaccessibility and catabolism of phenolic compounds from jaboticaba (*Myrciaria trunciflora*) fruit peel during in vitro gastrointestinal digestion and colonic fermentation. J. Funct. Foods.

[59] de Paulo Farias D., de Araújo F.F., Neri-Numa I.A., Dias-Audibert F.L., Delafiori J., Catharino R.R., Pastore G.M. (2021). Effect of in vitro digestion on the bioaccessibility and bioactivity of phenolic compounds in fractions of Eugenia pyriformis fruit. Food Res. Int..

[60] Mihaylova D., Desseva I., Stoyanova M., Petkova N., Terzyiska M., Lante A. (2021). Impact of in vitro gastrointestinal digestion on the bioaccessibility of phytochemical compounds from eight fruit juices. Molecules.

[61] Van de Velde F., Pirovani M.E., Drago S.R. (2018). Bioaccessibility analysis of anthocyanins and ellagitannins from blackberry at simulated gastrointestinal and colonic levels. J. Food Compos. Anal..

[62] Bešlo D., Golubić N., Rastija V., Agić D., Karnaš M., Šubarić D., Lučić B. (2023). Antioxidant Activity, Metabolism, and Bioavailability of Polyphenols in the Diet of Animals. Antioxidants.

[63] Odriozola-Serrano I., Nogueira D.P., Esparza I., Vaz A.A., Jiménez-Moreno N., Martín-Belloso O., Ancín-Azpilicueta C. (2023). Stability and bioaccessibility of phenolic compounds in rosehip extracts during in vitro digestion. Antioxidants.

[64] Lee J.E., Kim G.S., Park S., Kim Y.H., Kim M.B., Lee W.S., Jeong S.W., Lee S.J., Jin J.S., Shin S.C. (2014). Determination of chokeberry (*Aronia melanocarpa*) polyphenol components using liquid chromatography–tandem mass spectrometry: Overall contribution to antioxidant activity. Food Chem..

[65] Clifford M.N., Jaganath I.B., Ludwig I.A., Crozier A. (2017). Chlorogenic acids and the acyl-quinic acids: Discovery, biosynthesis, bioavailability and bioactivity. Nat. Prod. Rep..

[66] Naranjo Pinta M., Montoliu I., Aura A., Seppänen-Laakso T., Barron D., Moco S. (2018). In vitro gut metabolism of [U-13C]-Quinic acid, the other hydrolysis product of chlorogenic acid. Mol. Nutr. Food Res..

[67] Kasprzak-Drozd K., Oniszczuk T., Soja J., Gancarz M., Wojtunik-Kulesza K., Markut-Miotła E., Oniszczuk A. (2021). The Efficacy of Black Chokeberry Fruits against Cardiovascular Diseases. Int. J. Mol. Sci..

[68] Domínguez-Avila J.A., Wall-Medrano A., Velderrain-Rodríguez G.R., Chen C.Y.O., Salazar-López N.J., Robles-Sánchez M., GonzálezAguilar G.A. (2017). Gastrointestinal interactions, absorption, splanchnic metabolism and pharmacokinetics of orally ingested phenolic compounds. Food Funct..

[69] Wojtunik-Kulesza K., Oniszczuk A., Oniszczuk T., Combrzyński M., Nowakowska D., Matwijczuk A. (2020). Influence of In Vitro Digestion on Composition, Bioaccessibility and Antioxidant Activity of Food Polyphenols—A Non-Systematic Review. Nutrients.

[70] Salazar-López N.J., González-Aguilar G.A., Rouzaud-Sández O., Robles-Sánchez M. (2018). Bioaccessibility of hydroxycinnamic acids and antioxidant capacity from sorghum bran thermally processed during simulated in vitro gastrointestinal digestion. J. Food Sci. Technol..

[71] Bobrich A., Fanning K.J., Rychlik M., Russell D., Topp B., Netzel M. (2014). Phytochemicals in Japanese plums: Impact of maturity and bioaccessibility. Food Res. Int..

[72] Coman V., Vodnar D.C. (2020). Hydroxycinnamic acids and human health: Recent advances. J. Sci. Food Agric..

[73] Taofiq O., González-Paramás A.M., Barreiro M.F., Ferreira I.C. (2017). Hydroxycinnamic acids and their derivatives: Cosmeceutical significance, challenges and future perspectives, a Review. Molecules.

[74] Pavan V., Sancho R.A.S., Pastore G.M. (2014). The effect of in vitro digestion on the antioxidant activity of fruit extracts (*Carica papaya*, *Artocarpus heterophillus* and *Annona marcgravii*). LWT-Food Sci. Technol..

